# Transcriptomic changes underlying EGFR inhibitor resistance in human and mouse models of basal-like breast cancer

**DOI:** 10.1038/s41598-022-25541-3

**Published:** 2022-12-08

**Authors:** Narmeen S. Rashid, David C. Boyd, Amy L. Olex, Jacqueline M. Grible, Alex K. Duong, Mohammad A. Alzubi, Julia E. Altman, Tess J. Leftwich, Aaron D. Valentine, Nicole S. Hairr, Emily K. Zboril, Timothy M. Smith, Adam D. Pfefferle, Mikhail G. Dozmorov, J. Chuck Harrell

**Affiliations:** 1 Department of Pathology, Virginia Commonwealth University, Richmond, VA 23220 USA; 2 Department of Biology, University of Richmond, Richmond, VA 23173 USA; 3 Program in Integrative Life Sciences, Virginia Commonwealth University, Richmond, VA 23220 USA; 4 C. Kenneth and Diane Wright Center for Clinical and Translational Research, Virginia Commonwealth University, Richmond, VA 23220 USA; 5 Oncology Center-Division of Pediatric Oncology, Johns Hopkins University, Baltimore, MD 21287 USA; 6 Lineberger Comprehensive Cancer Center, University of North Carolina at Chapel Hill, Chapel Hill, NC 27514 USA; 7 Department of Genetics, University of North Carolina at Chapel Hill, Chapel Hill, NC 27514 USA; 8 Department of Biostatistics, Virginia Commonwealth University, Richmond, VA 23220 USA; 9 Massey Cancer Center, Virginia Commonwealth University, Richmond, VA 23220 USA

**Keywords:** Breast cancer, Breast cancer, Cancer genomics, Cancer therapy, Cancer therapeutic resistance, Cancer

## Abstract

The goals of this study were to identify transcriptomic changes that arise in basal-like breast cancer cells during the development of resistance to epidermal growth factor receptor inhibitors (EGFRi) and to identify drugs that are cytotoxic once EGFRi resistance occurs. Human patient-derived xenografts (PDXs) were grown in immunodeficient mice and treated with a set of EGFRi; the EGFRi erlotinib was selected for more expansive in vivo studies. Single-cell RNA sequencing was performed on mammary tumors from the basal-like PDX WHIM2 that was treated with vehicle or erlotinib for 9 weeks. The PDX was then subjected to long-term erlotinib treatment in vivo. Through serial passaging, an erlotinib-resistant subline of WHIM2 was generated. Bulk RNA-sequencing was performed on parental and erlotinib-resistant tumors. In vitro high-throughput drug screening with > 500 clinically used compounds was performed on parental and erlotinib-resistant cells. Previously published bulk gene expression microarray data from MMTV-Wnt1 tumors were contrasted with the WHIM2 PDX data. Erlotinib effectively inhibited WHIM2 tumor growth for approximately 4 weeks. Compared to untreated cells, single-cell RNA sequencing revealed that a greater proportion of erlotinib-treated cells were in the G1 phase of the cell cycle. Comparison of WHIM2 and MMTV-Wnt1 gene expression data revealed a set of 38 overlapping genes that were differentially expressed in the erlotinib-resistant WHIM2 and MMTV-Wnt1 tumors. Comparison of all three data types revealed five genes that were upregulated across all erlotinib-resistant samples: IL19, KLK7, LCN2, SAA1, and SAA2. Of these five genes, LCN2 was most abundantly expressed in triple-negative breast cancers, and its knockdown restored erlotinib sensitivity in vitro. Despite transcriptomic differences, parental and erlotinib-resistant WHIM2 displayed similar responses to the majority of drugs assessed for cytotoxicity in vitro. This study identified transcriptomic changes arising in erlotinib-resistant basal-like breast cancer. These data could be used to identify a biomarker or develop a gene signature predictive of patient response to EGFRi. Future studies should explore the predictive capacity of these gene signatures as well as how LCN2 contributes to the development of EGFRi resistance.

## Introduction

Triple negative breast cancer (TNBC) is an aggressive, highly metastatic breast cancer subtype that is characterized by a lack of hormone receptors and human epidermal growth factor receptor 2 (HER2)^1,2^. Thus, TNBC patients are not candidates for endocrine therapies or targeted therapy with anti-HER2 agents. TNBC patients face limited therapeutic options; chemotherapy is standard of care. TNBCs are a heterogeneous class and can be categorized into distinct subtypes: basal-like (1 and 2), claudin-low, immunomodulatory, mesenchymal-like, mesenchymal stem-like, and luminal androgen receptor positive^3,4^. These subtypes are transcriptionally distinct and display unique biology, immune composition, and sensitivity to chemotherapy^5^. Basal-like TNBCs are associated with the worst prognoses of all TNBC subtypes, indicating a need to identify efficacious treatment strategies for basal-like TNBC^6^. Drug resistance is a major clinical problem in basal-like TNBCs; we were interested in identifying strategies to overcome this clinical deficit by using targeted drugs, particularly EGFR inhibitors (EGFRi).

Basal-like breast cancers express relatively high levels of epidermal growth factor receptor (EGFR) compared to other breast cancer subtypes^7^. The EGFR family is composed of four categories of transmembrane tyrosine kinase receptors (ERBB1-4)^8^. Upon ligand binding, the inactive EGFR monomers dimerize to form active heterodimers. Dimerization is necessary for phosphorylation of the intracellular receptor kinase domain and activation of downstream pathways^8^. Once phosphorylated, EGFR can activate the PI3K/AKT and RAS signaling pathways. EGFRs are overexpressed and/or mutated in many cancers, including breast cancer^9^. EGFR overexpression or mutation can lead to aberrant signaling and promotion of uncontrolled cell growth and proliferation. EGFR overactivation in cancers is associated with poorer prognoses^9^. Currently, EGFRi are standard of care for patients with EGFR mutation-positive non-small cell lung cancer (NSCLC)^10^. However, the majority of patients treated with EGFRi for EGFR mutation-positive NSCLC will develop resistance to EGFRi^10^. Secondary mutations to the EGFR ligand binding domain, activation of compensatory pathways, and impairment of EGFR-EGFRi mediated apoptotic pathways are all mechanisms of resistance^11–13^. HGF overexpression, low BIM expression, PIK3CA mutations, and PTEN deletions have been associated with primary resistance to EGFRi^14^. In TNBC clinical trials, EGFRi have exhibited a modest response in combination with platinum compounds in a subset of patients, highlighting the need for predictors of therapeutic selection^15,16^.

PDXs have been shown to largely maintain the properties of the patient tumors from which they were derived^17^. Previous studies have found that basal-like PDXs have transcriptional profiles and metastasis patterns similar to patient samples within The Cancer Genome Atlas (TCGA) breast cancer cohort dataset^18^. In addition, several of these models have been found to be insensitive to chemotherapeutics^19,20^. In this study, we sought to identify a basal-like patient-derived xenograft (PDX) that was sensitive to EGFRi treatment, develop an EGFRi-resistant subline, and then, identify transcriptomic alterations underlying acquired resistance through bulk and single-cell RNA sequencing. Parallel analyses of transcriptomic data from isogenic transgenic mouse models of basal-like disease were incorporated to identify shared transcriptomic characters underlying EGFRi resistance. We also sought to identify drugs that demonstrate high levels of cytotoxicity in erlotinib-resistant basal-like PDXs via high-throughput drug screening. We hypothesize that these insights could be beneficial for (1) stratification of patients that could be responsive to EGFRi and (2) identification of effective therapies for patients with EGFRi-resistant disease.

## Methods

### In vivo growth of breast cancer PDX models

The following basal-like triple-negative breast cancer PDX models were used in this study: (HCI-001, UCD52, WHIM2). HCI-001 was obtained from the Huntsman Cancer Institute, University of Utah; WHIM2 was obtained from Washington University, St. Louis; UCD52 was obtained from the University of Colorado. All studies involving mice were approved by the Virginia Commonwealth University (VCU) Institutional Animal Care and Use Committee (IACUC) (Protocol# AD10001247), and all experiments were performed in accordance with IACUC guidelines and regulations, as well as the ARRIVE guidelines 2.0. Tumor fragments were grown in the mammary fat pads of female non-obese diabetic severe combined immunodeficient gamma (NSG) mice (The Jackson Laboratory, strain #005557). When tumors reached approximately 10 mm × 10 mm, the mice were euthanized with isoflurane anesthesia, cervical dislocation and thoracotomy, and the tumors were excised. Tumors were prepared into single-cell suspensions using a previously described protocol^21^. Single-cell suspensions were used for serial passaging by suspending tumor cells 1:1 in Matrigel (Corning) or Cultrex (Bio-Teche) and injecting tumor cells (500,000 cells per injection) into the mammary fat pads of mice. Single-cell suspensions were also used for in vitro drug screens.

### In vivo drug treatments

For the study in Supplementary Fig. 1, all drugs were dissolved in a solution of 1% methylcellulose + 0.1% Tween-80 and administered via oral gavage. Drugs were administered daily for 10 days: CO-1686 [100 mg/kg], erlotinib [100 mg/kg], gefitinib [200 mg/kg], dacomitinib [10 mg/kg], lapatinib [100 mg/kg], and afatinib [50 mg/kg]. For longer-term erlotinib treatment, mice received 367 ppm erlotinib-incorporated mouse chow (Envigo) ad libitum until a resistant phenotype arose.

### High-throughput drug screens

Single-cell suspensions of PDX cells were plated in 96-well plates at 16,000 cells per well in M87 medium and treated with 516 drugs (ApexBio DiscoveryProbe FDA-approved Drug Library) at 10 µM^22^. After 72 h, the CellTiter-Glo Luminescent Viability Assay (Promega) was used according to the manufacturer’s protocol. Cell viability was quantified by normalizing treated wells to vehicle (0.1% DMSO) wells to produce a percent of vehicle value. Drug cytotoxicity was compared between the parental WHIM2 and erlotinib-resistant WHIM2 PDXs. Three separate tumors were tested in duplicate, and replicates were then averaged for each PDX.

### Single-cell RNA sequencing

Single-cell transcriptomes were obtained from 1,314 parental tumor cells and 844 erlotinib-resistant tumor cells with a 10× Genomics Chromium single-cell controller utilizing a 10× Genomics Single Cell 3′Reagent kit standard protocol. Libraries were then sequenced on an Illumina Nextseq500/550 with 42-bp paired end reads, or a HiSeq2500 v4 with 125-bp paired end reads. The 10X Genomics CellRanger v6 software suite of tools was used to align samples and calculate gene expression. An in-house R script utilizing the Seurat v3.1.5 package was used to remove poor quality or dead cells. Additional filtering and realignment were performed to remove mouse cells. A final merged dataset containing only human cells was created using CellRanger. 10X Loupe Cell Browser v6.0.0 was used to visualize cell clusters and perform differential gene expression analyses across clusters^23^. Chi-squared test was performed using GraphPad Prism v.9.2.0 to identify if differences in the proportion of cells in each phase of the cell cycle were statistically significant between parental and erlotinib-treated tumors.

### Bulk RNA-sequencing

Parental WHIM2 (*n* = 4) and erlotinib-resistant WHIM2 (*n* = 3) mammary tumors tissues were excised and flash frozen. RNA was prepared with the Qiagen RNeasy mini kit. Sequencing libraries were prepared with NEBNext Ultra II RNA Library Prep Kit for Illumina using manufacturer’s instructions (New England Biolabs). The sequencing libraries were multiplexed and clustered onto a flowcell. After clustering, the flowcell was loaded onto the Illumina HiSeq instrument according to manufacturer’s instructions. The samples were sequenced using a 2 × 150 bp Paired End (PE) configuration. Image analysis and base calling were conducted by the HiSeq Control Software (HCS). Raw sequence data (.bcl files) generated from Illumina HiSeq was converted into fastq files and de-multiplexed using Illumina bcl2fastq 2.17 software. One mismatch was allowed for index sequence identification. Approximately 30 M reads were obtained per sample. Reads were aligned to a hg38 human reference genome. DEseq2 was utilized to identify fold change in gene expression and genes significantly differentially expressed in erlotinib-resistant WHIM2 tumors; *P* < 0.05 was considered statistically significant^24^. Gene Set Enrichment Analysis (GSEA) was performed to identify upregulated and downregulated genetic programs^25,26^.

### Ingenuity pathway analysis

Qiagen Ingenuity Pathway Analysis (IPA) was used to perform network analyses^27^. False discovery rate adjusted p-values (q-values) were calculated for bulk RNA-sequencing data to identify significantly differentially expressed genes in parental and erlotinib-resistant WHIM2 samples. IPA was performed on 228 genes meeting the following parameters: log experimental fold change > 2.0 and q-value < 0.05. Pathway analyses revealed upregulated and downregulated gene expression programs between samples.

### Immunohistochemistry

Immunohistochemical staining was performed on formalin-fixed, paraffin-embedded WHIM2 tumors. Heat-induced antigen retrieval was performed in pH 9 Tris–EDTA using a Dakocytomatin Pascal Pressure Chamber. EGFR (Cell Signaling Technology, 2232S) antibody was diluted 1:50 in SignalStain Antibody Diluent (Cell Signaling Technology) and was applied to sections from parental and erlotinib-resistant WHIM2 tumors. Detection was performed using the Rabbit Dako EnVision System (Agilent K406511–2). Slides were imaged using Zeiss Axio Observer with Zen software. Quantification of immunohistochemical staining was performed using ImageJ plugins IHC Toolbox and IHC Profiler^28^. T-tests were performed on GraphPad Prism v.9.2.0 to determine if differences in staining were statistically significant between groups.

### Wnt1-Early vs Wnt1-Late gene expression analysis

Gene expression microarray data from 37 untreated, FVB MMTV-*Wnt1* tumors were downloaded from the UNC Microarray Database as log2 Cy5/Cy3 ratios, filtering for probes with Lowess normalized intensity values greater than ten in both channels and for probes with data on greater than 70% of the microarrays^29,30^. After median centering the expression of each probe and imputing via the ten-nearest neighbor gene values, the dataset was collapsed by averaging the expression of probes corresponding to the same gene symbol. A two-class (Wnt1-Early vs Wnt1-Late) Significance Analysis of Microarrays (SAM) analysis was performed to identify differentially expressed genes^31^.

### Identification of 38 overlapping differentially expressed genes in basal-like erlotinib-resistant samples

DEseq2 identified 1641 significantly differentially expressed genes in erlotinib-resistant WHIM2 compared to parental WHIM2 (*P*_*adj*_ < 0.05). Of these genes, 521 had a fold change greater than or equal to 1.5. SAM analysis identified 9424 significantly differentially expressed genes in Wnt-Late compared to Wnt-Early (SAM q-value < 5%). Of these genes, 2833 genes had a fold change greater than or equal to 1.5. Significantly differentially expressed genes with a fold change ≥ 1.5 in erlotinib-resistant WHIM2 and Wnt-Late were compared. There were 92 overlapping genes. Of these genes, 38 were upregulated or downregulated in the same direction in erlotinib-resistant WHIM2 and Wnt-late samples. Heatmaps depicting differential gene expression were created using Morpheus (https://software.broadinstitute.org/morpheus/). Data were hierarchically clustered by both samples (erlotinib-sensitive vs resistant) and genes using the one minus Pearson correlation metric and average linkage method. Boxplots of TCGA data were generated with http://ualcan.path.uab.edu/index.html^32,33^.

### Transient knock-down of LCN2 using siRNA lipofection

WHIM2 erlotinib-resistant cells were harvested and transfected per the manufacturer’s protocol. For the experimental group, siGENOME Human LCN2 siRNA SMARTPool (Dharmacon) was utilized, which contained four targeting siRNAs for the LCN2 transcript. For the control group, a non-targeting siGENOME siRNA control pool (Dharmacon) of four siRNAs were used. Both siRNAs and DharmaFECT 1, a lipo-based transfection reagent, were diluted into serum-free, antibiotic-free media separately and shortly incubated. Then, for each group, the siRNA and DharmaFECT media were plated together in 100 mm dishes for another incubation period. Cells were suspended in antibiotic-free media containing serum, plated on top of the siRNA and DharmaFECT media solution, and gently mixed. Optimization led to a transfection period of 1 day. Transfection was ended by replacing transfection media with M87 media. Knock down was confirmed via western blot analysis.

### Ethical approval and consent to participate

All animal and cell line work were done according to VCU Institutional Animal Care and Use Committee protocols.

## Results

### Select EGFR inhibitors reduced the growth of the WHIM2 PDX

In previous studies, the WHIM2 basal-like PDX was not responsive to the chemotherapeutic carboplatin^19,20^; however, several different EGFRi demonstrated cytotoxic activity in vitro^21^. To identify EGFRi that were effective in vivo, NSG mice bearing palpable WHIM2 mammary tumors were treated daily with one of six different EGFRi: CO-1686, erlotinib, gefitinib, dacomitinib, lapatinib, or afatinib. At the concentrations tested, erlotinib, gefitinib, dacomitinib, and afatinib each prevented tumor growth over a 10-day period (Supplementary Fig. 1). Since erlotinib is FDA-approved for clinical use and has a well-characterized side effect profile, it was chosen as the EGFRi to be used for the remainder of the study. A separate cohort of WHIM2 tumor-bearing mice was then treated with erlotinib-incorporated mouse chow, which also successfully inhibited tumor growth (Supplementary Fig. 1).

### Development of an acquired erlotinib-resistant PDX model

**Figure 1:**
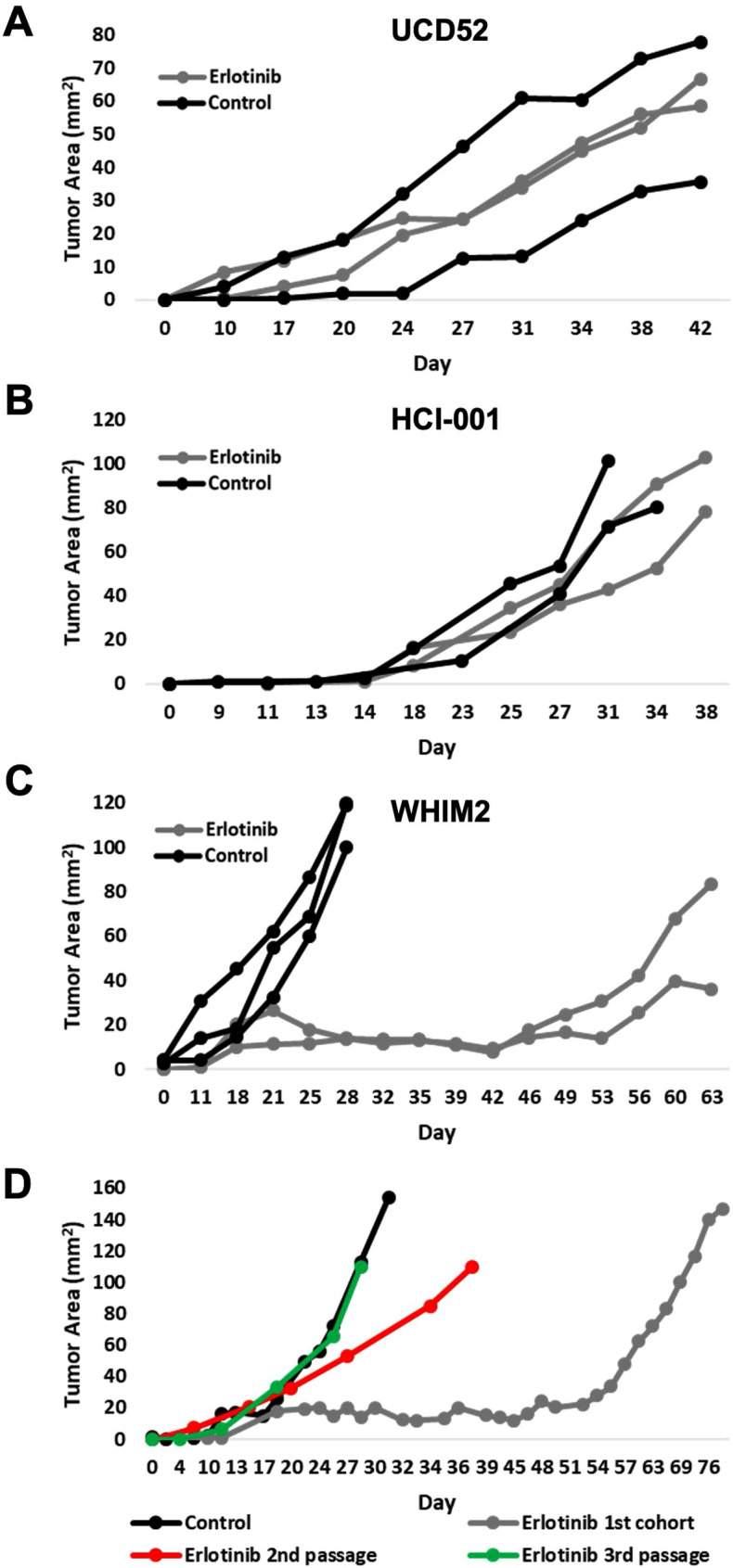
Development of acquired erlotinib-resistance in an erlotinib-sensitive PDX. (**A**) UCD52; (**B**) HCI-001; and (**C**) WHIM2 PDXs were treated with erlotinib-incorporated mouse chow ad libitum once tumors were palpable; (**D**) WHIM2 tumors were treated with erlotinib chow until resistance arose (1st passage) (*n* = 2). The first cohort of resistant tumors were passaged into a second cohort of mice (2nd passage) (*n* = 4), and mice were treated with erlotinib chow once tumors were palpable. The second cohort’s tumors were then passaged into a third cohort of mice and treated with erlotinib chow. The third cohort’s tumors were considered erlotinib-resistant (*n* = 10).

The tumor growth inhibitory activity of erlotinib was next assessed across two additional basal-like PDXs: UCD52 and HCI-001; however, no significant effect on tumor growth was observed in these models (Fig. 1A,B). Longer-term treatment of the WHIM2 PDX found that erlotinib effectively prevented tumor growth for at least 4 weeks before tumor growth resumed (Fig. 1C). Drug-resistant tumors were serially passaged into new recipient mice (2nd passage) which were then treated with erlotinib chow. This process was repeated a second time (3rd passage), and the tumor growth rate was similar to the parental WHIM2 (Fig. 1D). The erlotinib-resistant PDX was termed WHIM2/ErlR.

### Identification of compounds that were cytotoxic to WHIM2/ErlR cells

**Figure 2:**
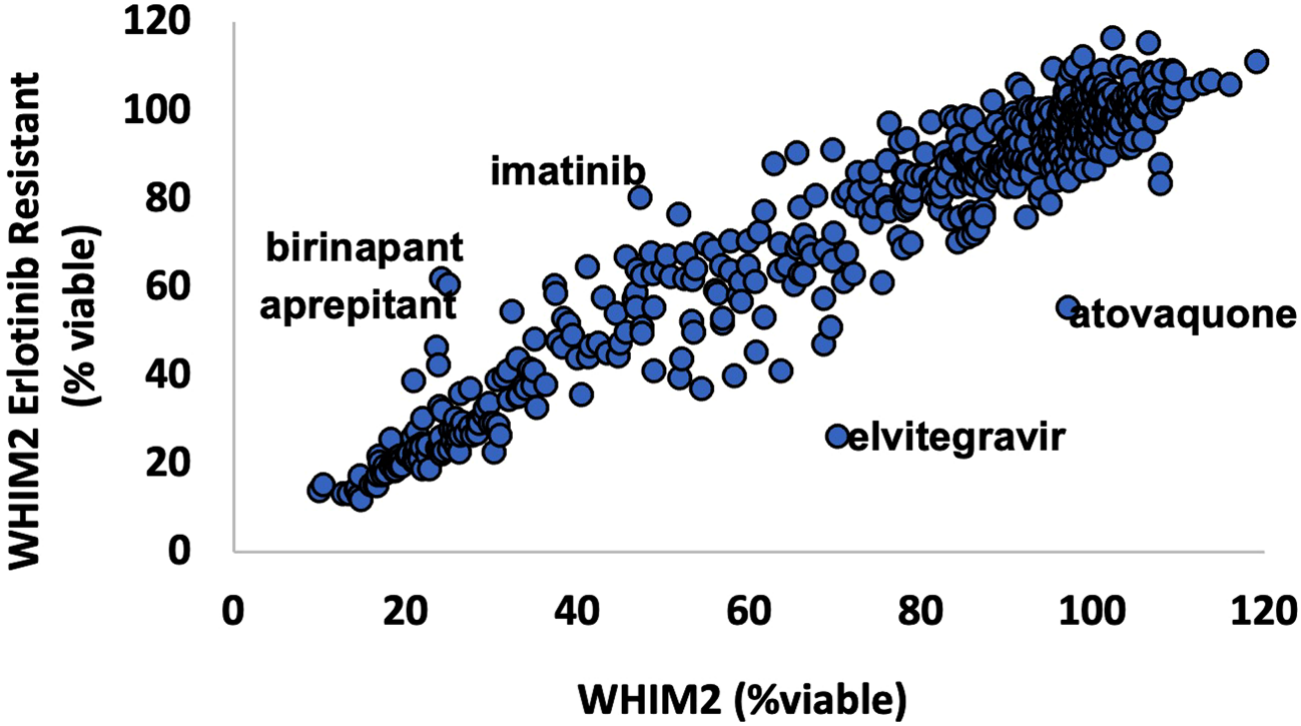
Assessment of 516 FDA-approved and clinically used drugs on parental and erlotinib-resistant WHIM2 PDXs. Tumors were excised from mice, prepped into single cell suspensions, plated in 96-well dishes, and treated with 10 μM of drug. Cell viability was assessed with CellTiter-Glo after 72 h of treatment and normalized to DMSO vehicle control. Parental (*n* = 3) and erlotinib-resistant (*n* = 3) tumors were treated in triplicate.

When primary or secondary drug resistance occurs in the clinical setting, new therapeutic approaches are needed. Therefore, 516 clinically-utilized compounds were individually tested for cytotoxic activity on single-cell suspensions obtained from WHIM2 or WHIM2/ErlR cells. Interestingly, the majority of compounds displayed similar efficacy on both the parental and WHIM2/ErlR cells (Fig. 2). Notable exceptions that demonstrated greater efficacy on WHIM2/ErlR cells included elvitegravir (GS-9137), an integrase inhibitor used to treat human immunodeficiency virus infection, and atovaquone, a quinone antimicrobial. Conversely, several drugs demonstrated reduced efficacy on the WHIM2/ErlR cells, including birinapant, aprepitant, and imatinib. Importantly, most of the highly cytotoxic drugs resulted in cell death to both parental and WHIM2/ErlR cells. Examples include topoisomerase inhibitors (doxorubicin, idarubicin, epirubicin), proteasome inhibitors (MLN2238/Ixazomib, CEP-18770, carfilzomib), HDAC inhibitors (belinostat, PCI-24781), and other EGFR inhibitors (neratinib, dacomitinib, afatinib), among others.

### Single-cell transcriptional responses to erlotinib treatment

**Figure 3:**
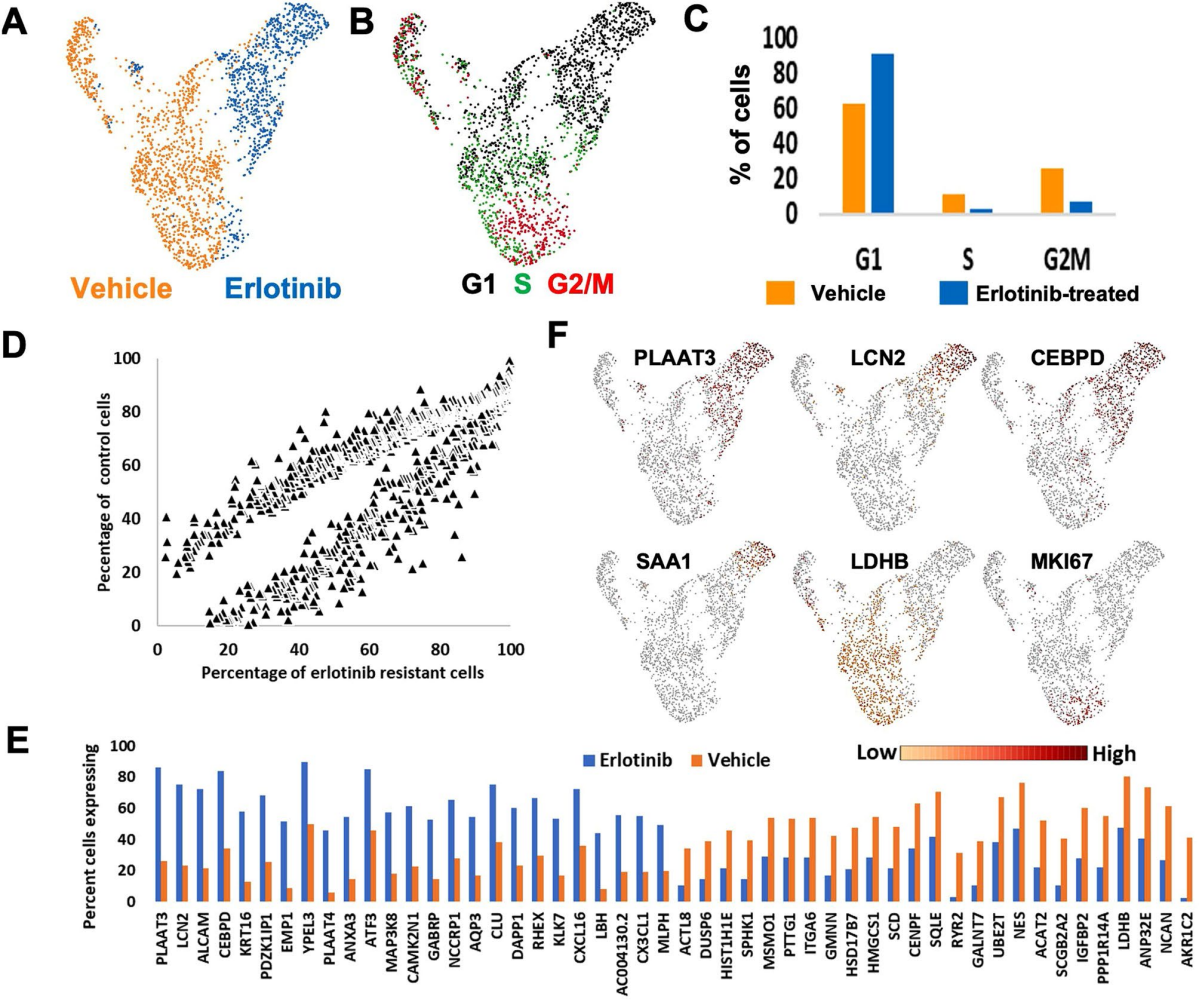
Single-Cell RNA sequencing identified subpopulations that mediate erlotinib resistance. Uniform Manifold Approximation and Projection (UMAP) plots of scRNA-seq data from vehicle or erlotinib-treated WHIM2 tumors. UMAP plots are two-dimensional plots depicting clusters of specific cell types (e.g. vehicle vs erlotinib-treated). UMAP plots are colored based on (**A**) treatment status or; (**B**) cell cycle status as assessed through cell cycle phase-specific gene signatures; (**C**) The proportion of cells from the vehicle tumor and erlotinib-treated tumor in each phase of the cell cycle, as shown in plot B; (**D**) Plotted are 1,103 transcripts identified as significantly differentially expressed between treatment conditions. Each axis shows the percentage of cells in each treatment condition that expressed the RNA transcript; (**E**) Plot depicting the 25 most differentially upregulated genes each treatment condition, as determined by the percentage of cells in the sample expressing each transcript; (**F**) UMAP plots depicting examples of genes that were differentially expressed following erlotinib treatment; MKI67 is shown to label G2/M cells.

Single-cell RNA-sequencing (scRNA-seq) was used to identify transcriptional changes that occurred at the single-cell level upon the development of erlotinib resistance in the WHIM2 PDX. Mice with palpable WHIM2 PDX tumors were treated with erlotinib until drug-resistance occurred (9 weeks). Treated and control tumors were prepared into single-cell suspensions, and scRNA-seq was performed. In total, 1,314 control cells and 844 treated cells were analyzed. Uniform Manifold Approximation and Projection (UMAP) based images of all cells were developed based on transcriptomic data from all significantly differentially expressed genes (Fig. 3A). Cell cycle phase specific gene expression signatures were assessed for each cell to determine if there were differences in the proportion of cells in each phase of the cell cycle due to treatment (Fig. 3B). Chi-squared test revealed a significant association between erlotinib treatment status and proportion of cells in each phase of the cell cycle (Χ^2^ = 208.6, df = 2, *P* < 0.0001). In the erlotinib-treated sample, there was an increase in the proportion of cells that were identified in the G1 cell state compared to the untreated sample (Fig. 3C). Conversely, there was a decrease in the proportion of cells that were identified in the S and G2M cell state in the erlotinib-treated sample compared to the untreated sample. Next, we identified differentially expressed genes which could have mediated erlotinib-resistance. Gene expression was quantified as percentage of cells in the sample expressing the gene transcript. There were 713 unique gene transcripts that were more abundantly expressed in the WHIM2 erlotinib-treated cells than in the WHIM2 parental cells; 390 other transcripts were more abundantly expressed in the parental cells than the erlotinib-treated cells (Fig. 3D). The 25 most differentially upregulated genes in the erlotinib-treated or control cells are shown (Fig. 3E). Examples of genes with more expression per cell in the erlotinib-treated group are PLAAT3, LCN2, CEBPD, and SAA1; conversely, LDHB is shown as an example for transcripts more abundant in control tumor cells. MKI67 is shown as a marker of the proliferating-G2/M population (Fig. 3F).

### Bulk RNA-seq analysis of erlotinib-resistant WHIM2 tumor cells

**Figure 4:**
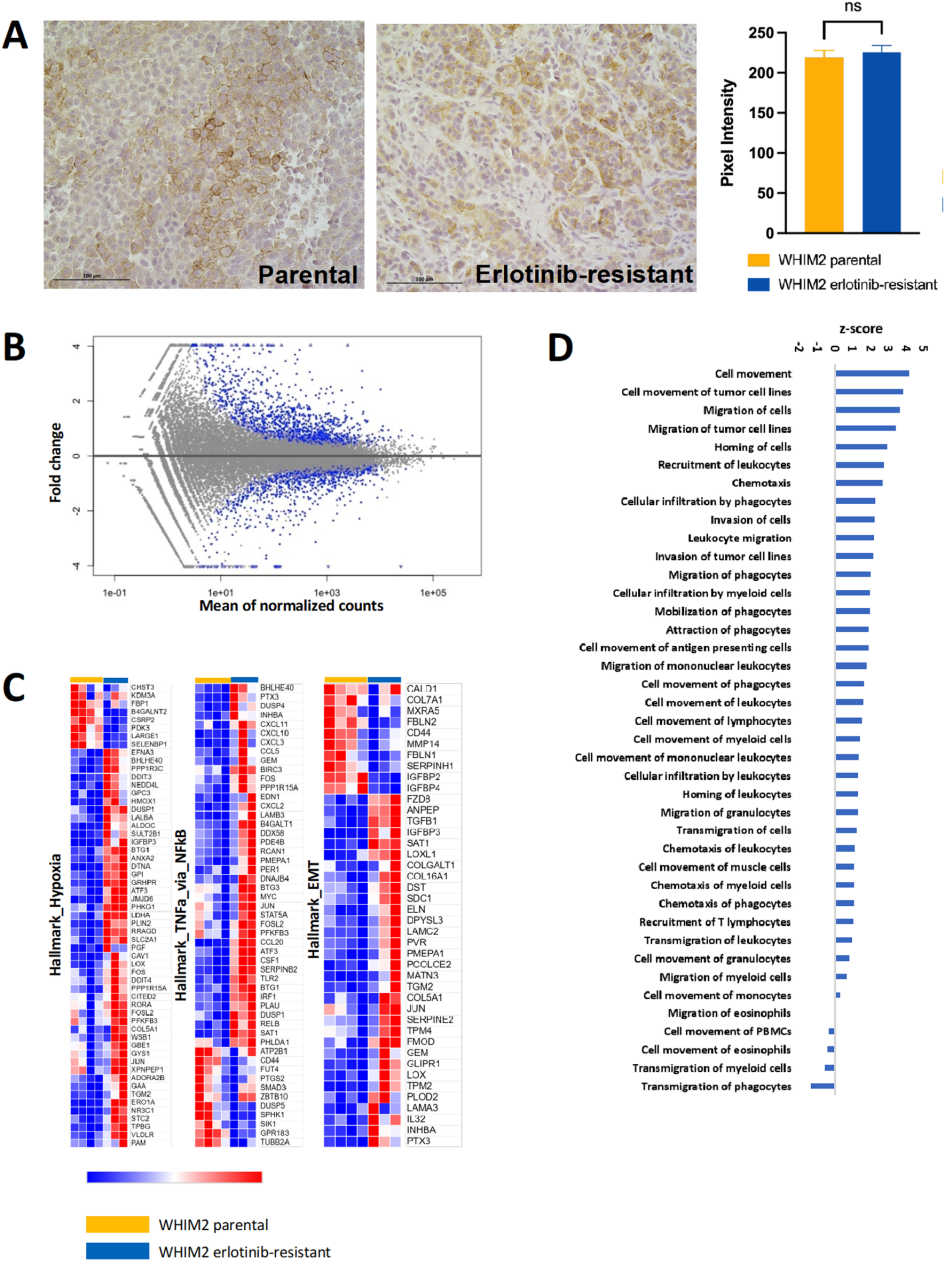
Assessment of erlotinib-resistance mechanisms in the WHIM2 PDX. (**A**) Immunohistochemical staining for EGFR on formalin-fixed, paraffin-embedded parental and erlotinib-resistant WHIM2 tumors and comparison of mean staining intensity of parental and erlotinib-resistant WHIM2 tumors (*P* = 0.41); (**B**) Each point represents a single gene. Plot depicts fold change in gene expression in erlotinib-resistant tumors relative to vehicle tumors. Blue points represent genes that were significantly differentially expressed in the erlotinib-resistant WHIM2 tumor (*P* < 0.05); (**C**) Examples of gene signatures upregulated in erlotinib-resistant tumors as determined by gene set enrichment analysis. Hallmark gene signatures for hypoxia, TNF-α via NFκB, and epithelial to mesenchymal transition are depicted; (**D**) Ingenuity Pathway Analysis identified significantly up- and down-regulated cellular gene expression programs in the WHIM2 erlotinib resistant cells.

After the WHIM2/ErlR subline was generated via serial passaging of erlotinib-treated tumors, immunohistochemical staining for EGFR was performed on parental WHIM2 and WHIM2 Erl/R tumors. Both were found to heterogeneously express EGFR (Fig. 4A). This suggests that selection for EGFR-negative cells was not the mechanism of resistance, nor was the gross upregulation of EGFR to compensate for its inhibition. Bulk RNA-sequencing data was then generated from the parental and WHIM2/ErlR PDXs. There were 521 transcripts identified as differentially expressed in the WHIM2/ErlR compared to WHIM2 parental (*P* < 0.05) (Fig. 4B). GSEA found that hallmark genetic programs of hypoxia, TNF-α signaling via NFκB, and epithelial mesenchymal transition were activated (Fig. 4C). Ingenuity Pathway Analysis was used to perform network analyses. Activation at p-value < 0.001 and z-score > 2.0 was predicted in 40 functions related to cellular movement in the WHIM2/ErlR (Fig. 4D). This suggests that cellular movement gene expression programs were upregulated in the development of erlotinib resistance in WHIM2.

### Comparison of the WHIM2 and MMTV-Wnt1 transgenic models

**Figure 5:**
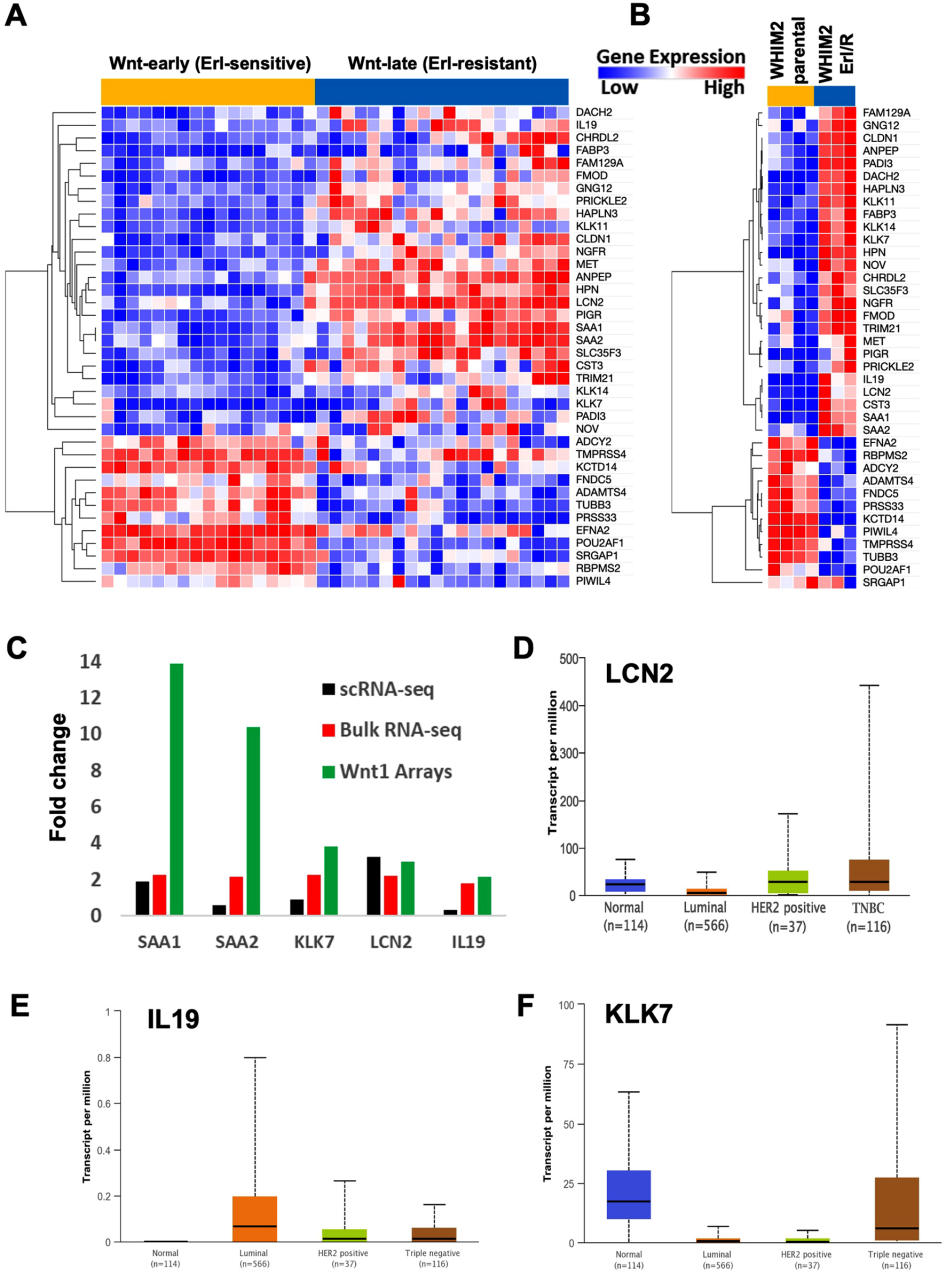
An overlapping set of 38 differentially expressed genes correlated with erlotinib response was identified within the Wnt1 transgenic model and the WHIM2 PDX model. Heat maps depicting the set of 38 overlapping genes within the (**A**) Wnt1 tumors and; (**B**) WHIM2 PDX tumors; (**C**) Five genes that were upregulated in all 3 types of data analyzed; (**D**–**F**) Plots depicting expression of the five genes found to be upregulated in all 3 data types were created using The Cancer Genome Atlas and UALCAN. Plots depict gene expression by major breast cancer subtype; plots depicting the three genes that were expressed in TNBC are shown.

The MMTV-Wnt1 transgenic basal-like mouse model spontaneously generates mammary tumors in a bi-modal distribution, either early (6.5 weeks) or late (22.5 weeks). Interestingly, the Wnt1-Early and Wnt1-Late tumors are transcriptionally distinct and respond differently to erlotinib^29^.Genomic differences between these isogenic tumors were contrasted with the WHIM2 parental and WHIM2/ErlR tumors. In total, 38 shared genes were found to be upregulated or downregulated during the development of erlotinib-resistance in the WHIM2 PDX; each of these genes was also increased or decreased in the same direction in the Wnt1-Late (erlotinib-resistant) and WHIM2/ErlR models (Fig. 5A,B). Five of these genes were also found to be significantly differentially expressed in the same direction in the scRNA-seq data (Fig. 5C). Analysis of TCGA breast cancer cohort dataset found that three of the genes were expressed in triple-negative breast cancers^33^ (Fig. 5D–F); LCN2 showed high transcriptional expression level in some tumors, similar to the above transcriptomic analyses.

### Differential LCN2 protein expression between parental and erlotinib-resistant tumors

**Figure 6:**
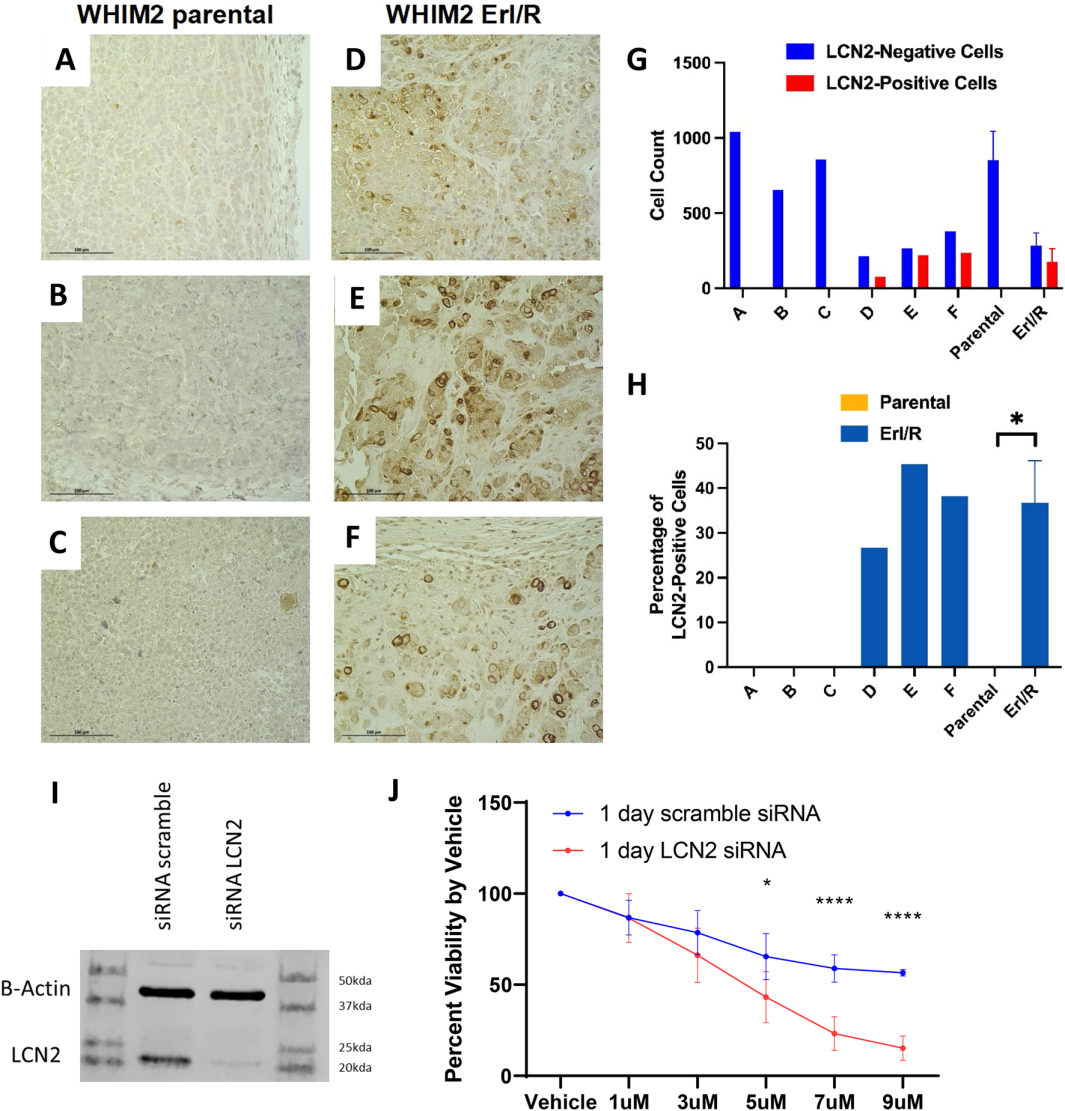
(**A**–**F**) Representative images of immunohistochemical staining for LCN2 on formalin-fixed, paraffin-embedded WHIM2 parental and WHIM2 Erl/R mammary gland tumors; (**G**) Quantification of LCN2-negative cells and LCN2-positive cells in each image; (**H**) Percentage of total cells in the image that were LCN2-positive (*P* = 0.021). (**I**) Western blot of lysates from LCN2 siRNA and non-targeting siRNA transfected WHIM2 erlotinib-resistant PDX cells. (**J**) Erlotinib treatment on WHIM2 erlotinib-resistant PDX cells with LCN2 siRNA or non-targeting siRNA transfection (*P* < 0.05 at 5 μM, *P* < 0.001 at 7 μM and 9 μM).

Since LCN2 was found to be highly expressed in a subset of TNBCs, immunohistochemical staining for LCN2 was performed on parental (erlotinib-sensitive) and Erl/R WHIM2 tumors to assess differential LCN2 protein expression. Compared to staining for EGFR, LCN2 expression appeared bimodal and less diffuse, in that each cell either expressed or did not express LCN2 protein. Consistent with the transcriptomic data, immunohistochemical staining of parental and Erl/R WHIM2 tumors found a significantly greater proportion of LCN2 positive cells in Erl/R tumors compared to parental tumors (*P* < 0.05) (Fig. 6A–H). To investigate LCN2’s role in erlotinib resistance, LCN2 siRNA transfection was used to knock down LCN2 expression in WHIM Erl/R PDX cells using non-targeting siRNAs for a control. Relative LCN2 protein was visualized via western blot (Fig. 6I). Transiently transfected cells were treated with 1, 3, 5, 7, and 9 µM of erlotinib in vitro. At 5, 7, and 9 µM, erlotinib demonstrated significantly greater cytotoxicity towards LCN2 siRNA transfected cells compared to non-targeting siRNA transfected cells (Fig. 6J).

## Discussion

In these studies, we sought to identify transcriptomic changes that accompany the development of EGFRi resistance in basal-like TNBC PDXs in order to identify biomarkers and gene signatures predictive of EGFRi response. In pilot studies, we identified an EGFRi that demonstrated in vivo antitumor activity in the WHIM2 basal-like TNBC PDX. Of these EGFRi, erlotinib was chosen for further study. We derived an erlotinib-resistant WHIM2 PDX from the parental PDX. ScRNA-seq and bulk-RNA sequencing were performed to identify transcriptomic changes underlying the development of erlotinib resistance. GSEA and IPA utilizing bulk-RNA sequencing data identified increased epithelial-to-mesenchymal transition (EMT) and cell movement, respectively, in WHIM2 Erl/R compared to parental WHIM2. This suggests that EGFRi resistance may be associated with genomic programs that are also increased during metastasis. Furthermore, drugs targeting EMT and cell movement may be effective in the restoring EGFRi sensitivity.

Further analysis of bulk-RNA sequencing data from WHIM2 and MMTV-Wnt1 transgenic mouse models identified 38 genes that were differentially expressed in erlotinib-resistant strains. Of the upregulated genes, five genes were also significantly upregulated in scRNA-seq. These genes included: SAA1, SAA2, KLK7, LCN2, and IL19. These genes could potentially serve as predictive biomarkers of erlotinib response. Interestingly, López-Ayllón et al. (2015) identified sixteen genes that were upregulated in erlotinib-resistant NSCLC tumors compared to erlotinib-sensitive tumors^34^. These genes included LCN2, MET, PIGR, and SAA1, all of which were found to be upregulated in WHIM2/ErlR and Wnt-late tumors compared to WHIM2 parental and Wnt-early tumors. Interestingly, many of the activated genes found within the model system presented herein and in these previous studies encode for proteins within inflammatory and natural immunity processes, suggesting that these biological processes are contributing to drug resistance. Krysan et al. also found that LCN2 overexpression was associated with erlotinib-resistance in NSCLC^35^.

Of the five genes found to be upregulated in all three data types analyzed, LCN2 was the only gene found to be overexpressed in TNBC. Previous studies have found a correlation between LCN2 expression and disease aggression. For example, LCN2 has been positively associated with tumorigenesis, invasiveness, migration, metastasis^36–39^. Interestingly, LCN2 was included in several IPA cell movement gene signatures found to be upregulated in the EGFRi resistant setting. This suggests that LCN2 may contribute to observed increases in EMT, cell movement, and metastasis observed in the EGFRi resistant setting.

**Figure 7:**
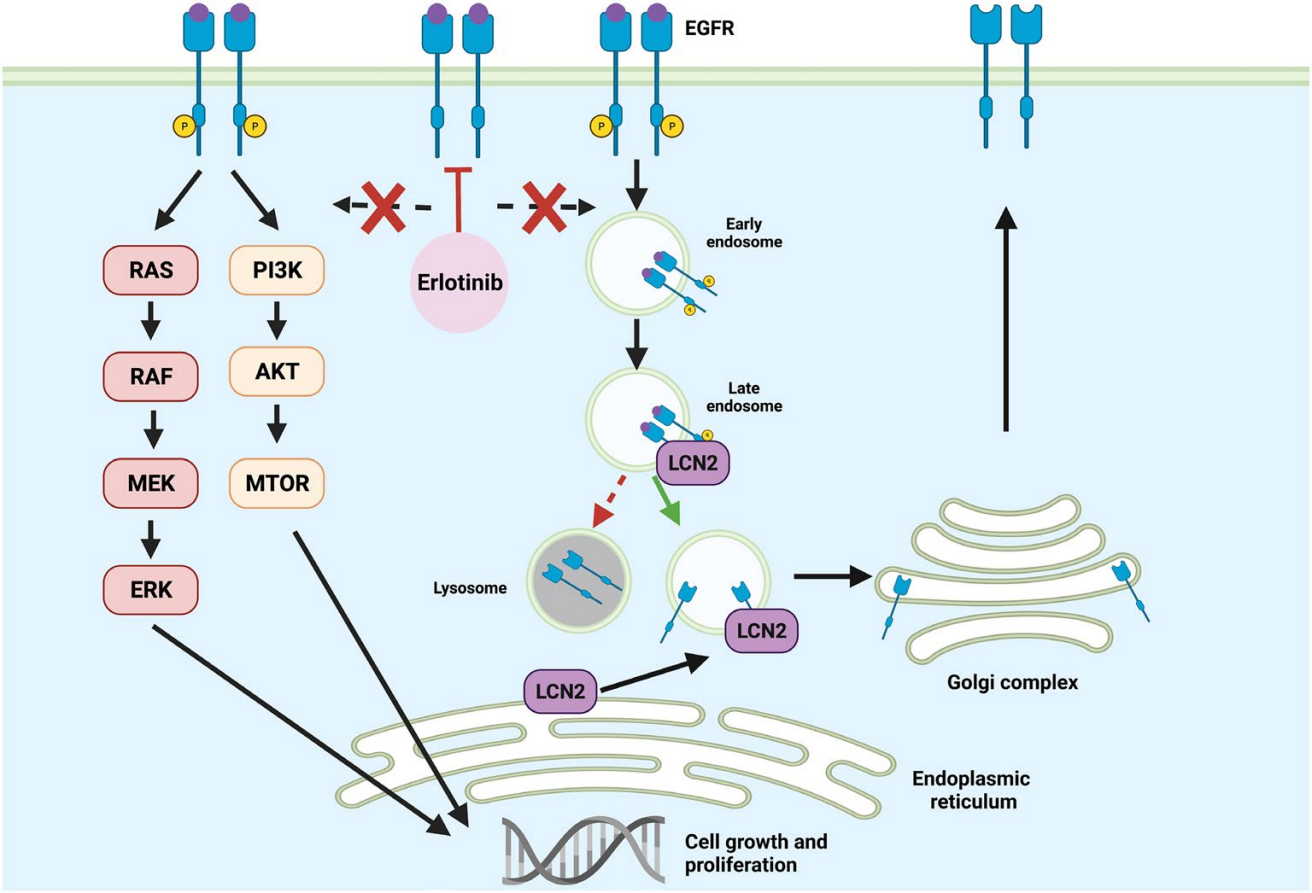
Hypothetical model depicting how LCN2 upregulation in erlotinib-resistant tumors may contribute to EGFR recycling back to the plasma membrane and allow for continued oncogenic EGFR pathway activity despite EGFR inhibition by erlotinib. Schematic of EGFR recycling via LCN2 was adapted from Yammine et al.^40^. Created with BioRender.com.

The mechanism by which LCN2 may contribute to acquired EGFRi resistance requires further study, but it may be related to EGFR recycling. Yammine et al. demonstrated that LCN2 increases EGFR abundance on the cell membrane^40^. They also found that LCN2 is involved in intracellular trafficking of EGFR and promotes recycling of EGFR to the plasma membrane. In this way, LCN2 upregulation may serve to salvage EGFR signaling in the presence of an EGFRi by returning EGFRs to the plasma membrane for further stimulation and activation of downstream pathways (Fig. 7). Previous analysis of MMTV-Wnt tumors revealed EGFR pathway amplification in Wnt-late tumors compared to Wnt-early tumors^29^. LCN2 upregulation may allow for EGFR pathway amplification via recycling of EGFRs and thereby promote resistance to EGFRi. When LCN2 was knocked down in Erl/R WHIM2 PDX cells in vitro, cells were more responsive to EGFRi. This suggests that LCN2 causally contributes to acquired EGFRi resistance, at least in the WHIM2 model of basal-like disease.

Despite transcriptomic differences, however, the WHIM2 parental and WHIM2/ErlR demonstrated similar responses to a panel of drugs including topoisomerase inhibitors, proteasome inhibitors, HDAC inhibitors, and other EGFR inhibitors. Notable exceptions were elvitegravir and atovaquone, both of which demonstrated greater cytotoxicity in WHIM2/ErlR than WHIM2 parental. Interestingly, several drugs demonstrated decreased efficacy in the WHIM2/ErlR line, including birinapant (SMAC inhibitor) and imatinib (ABL inhibitor). These data suggest that, in general, drugs that demonstrate high cytotoxicity in erlotinib-sensitive models are also cytotoxic towards erlotinib-resistant models.

Although basal-like tumors exhibit high levels of EGFR expression, EGFRis have only demonstrated modest antitumor activity in a subset of TNBC patients^15,16^. Through these studies, we found a set of genes that could potentially serve as predictive biomarkers of erlotinib response, and more generally, EGFRi response in basal-like TNBCs. This gene set could be used to identify patients that would best benefit from EGFRi. Future studies should evaluate the predictive capability of the described gene signatures, as well as how these genes contribute to the development of EGFRi resistance. Future studies should also focus on the identification of drugs that demonstrate increased cytotoxicity towards EGFRi-resistant models, as well as drugs that may re-sensitize EGFRi-resistant cells to EGFRi.

## Supplementary Information

Supplementary Figure 1.

## Data Availability

Single-cell RNA-sequencing: GSE189324. WHIM2 bulk RNA-sequencing: GSE189257. SuperSeries (links to both of the above): GSE189325. Wnt1 gene expression microarray data: GEO GPL11383. Other data are available from the corresponding author upon reasonable request.
