# Supplementary material for: Transcriptomic changes underlying EGFR inhibitor resistance in human and mouse models of basal-like breast cancer
Source: Sci Rep. 2022 Dec 8;12:21248. doi: 10.1038/s41598-022-25541-3 (PMC9731984; doi:10.1038/s41598-022-25541-3)
Supplement: Supplementary file 1 — Supplementary Figure 1. [file 41598_2022_25541_MOESM1_ESM.docx]

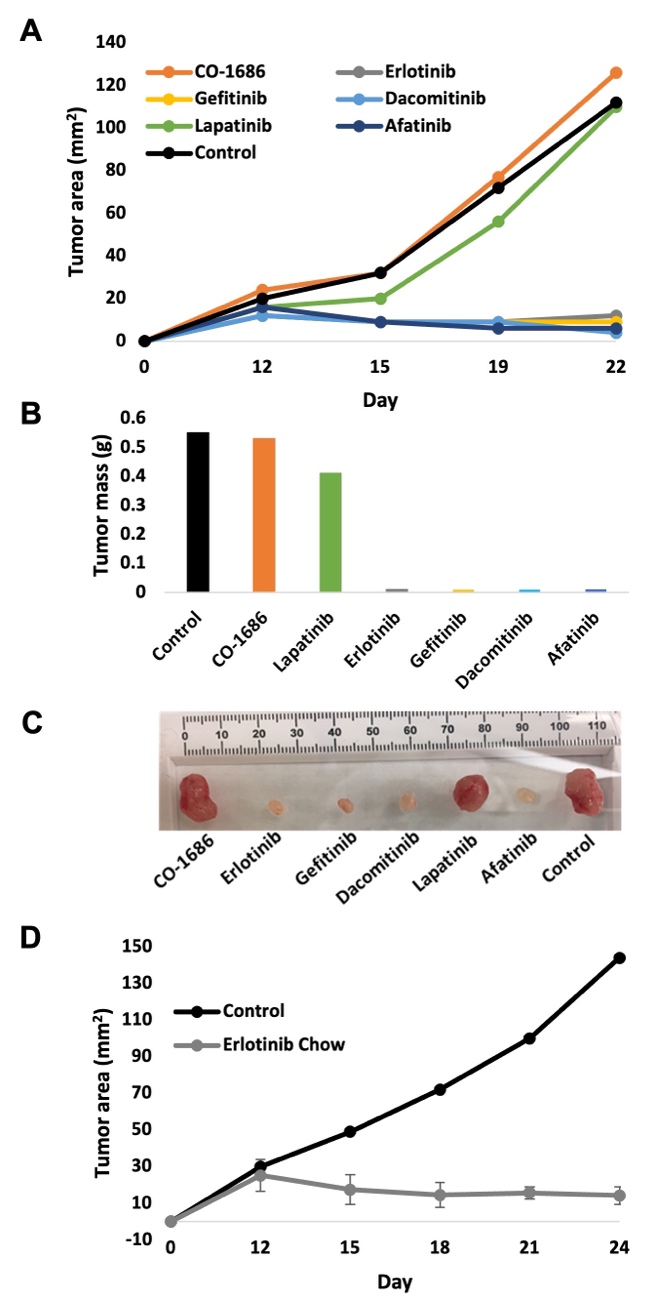


**Supplementary Figure 1.** Assessment of EGFR inhibitors on growth of the basal-like patient-derived xenograft WHIM2. Effect of daily oral gavage treatment with various EGFR inhibitors (*n* = 1 per drug): CO-1686 [100mg/kg], erlotinib [100mg/kg], gefitinib [200mg/kg], dacomitinib [10mg/kg], lapatinib [100mg/kg], afatinib [50mg/kg]. A: Tumor size; B: Excised tumor mass; C: Picture of excised tumors; D: A second cohort of mice was treated with erlotinib-incorporated mouse chow (367ppm) *ad libitum* (*n =* 1 control, *n* = 4 erlotinib treated). Tumor size over the course of treatment is depicted.
